# The Current Landscape of Bioactive Molecules against DENV: A Systematic Review

**DOI:** 10.1155/2023/2236210

**Published:** 2023-02-10

**Authors:** Ritchu Babbar, Ramanpreet Kaur, Parteek Rana, Sandeep Arora, Tapan Behl, Mohammed Albratty, Asim Najmi, Abdulkarim M. Meraya, Hassan A. Alhazmi, Rajeev K. Singla

**Affiliations:** ^1^Chitkara College of Pharmacy, Chitkara University, Chandigarh, Punjab, India; ^2^Department of Pharmaceutical Sciences, Amity University, Noida, India; ^3^School of Health Sciences, University of Petroleum and Energy Studies, Dehradun, Uttarakhand, India; ^4^Department of Pharmaceutical Chemistry and Pharmacognosy, College of Pharmacy, Jazan University, Jazan, Saudi Arabia; ^5^Pharmacy Practice Research Unit, Department of Clinical Pharmacy, College of Pharmacy, Jazan University, Jazan, Saudi Arabia; ^6^Department of Pharmaceutical Chemistry, College of Pharmacy, Jazan University, Jazan, Saudi Arabia; ^7^Substance Abuse and Toxicology Research Centre, Jazan University, Jazan, Saudi Arabia; ^8^Institutes for Systems Genetics, Frontiers Science Center for Disease-Related Molecular Network, West China Hospital, Sichuan University, Xinchuan Road, Chengdu, Sichuan, China; ^9^School of Pharmaceutical Sciences, Lovely Professional University, Phagwara, Punjab 144411, India

## Abstract

With a 30-fold increase in incidence over the previous 50 years, dengue fever is now the most widespread viral disease transmitted by mosquitoes in the world. The intricate interaction of the human defense system, hereditary predisposition, and specific bitterness elements is more likely to be the pathogenesis of dengue. There are presently no viable treatments for dengue. Synthetic drugs which are used against this ailment also show major side effects. There must be a deeper understanding of the underlying mechanism generating severe symptoms to develop auguring markers, cutting-edge diagnostics, and treatments and finally a well-rounded and secure antiserum. Hence, the aim is to search for safer and more potent drugs derived from plants. Plants or herbs are mainly targeting replication or its enzyme or specific stereotypes, though an exact mechanism of phytoconstituents interfering with the viral replication is still undiscovered. The present attempt provided the update with the objective to bringing up forward pathophysiological eventualities involved in dengue virus along with the naturally derived treatment relevant to provide the impregnable therapy by evading the noxious symptoms for dengue fever. *Governor's plum*, *Cryptocarya chartacea*, magnolia berry, and Chinese ginger are such plants exhibiting many effective phytoconstituents against DENV and can be further explored for novel drug discovery by medicinal scientists.

## 1. Introduction

The *Flaviviridae* class of virus that causes dengue fever, disseminated by mosquitoes, recently becomes widespread throughout all WHO zones. Over the past 20 years, dengue fever cases have doubled since 2000, when there were only 505,430 cases; by 2020 there were 5 million cases. Between 2000 and 2015, the reported death toll rose from 960 to 4032, with young people carrying most of the burden [1]. Any serotype of the dengue virus might result in dengue fever (DENV-1, DENV-2, DENV-3, and DENV-4), and future defense response against these virus types is typically brought on by infection with one type but not against others. In addition, if exposed to alternative type a second time, a more severe illness could manifest. This is brought on by a phenomenon called “immune subordinate upgrading” in which antibodies to one serotype enhance the contamination with a different serotype [2]. The four dengue virus serotypes DENV (1–4) are related to one another and have gene sequence similarities of 65 to 70 percent [3]. Virus dwells of lipid and polysaccharide envelope and 11 KB plus-strand RNA genome, three structural proteins-Glycoprotein, protein coat (C), membrane (M), and casing as well as seven nonstructural proteins:NS1, NS2A, NS2B, NS3, NS4A, NS4B, and NS5 [4]. Individual functions of these nonstructural proteins include NS1, responsible for viral infection. On the other side, RNA replication is aided by the lipophilic integral biological membrane peptide NS2A, while NS2B is a protease coenzyme. In viral RNA production, NS3 regulates the nucleoside triphosphate and helicase enzymes. NS4A aids in the formation of replication vesicles, while NS4B inhibits interferon beta and gamma signaling [5]. NS5 (105 KDa), the biggest and most widespread polypeptide, is necessary for RNA production and successfully inhibits the interferon (IFN) system [6]. Female mosquitoes exposed to the virus, generally the *Aedes aegypti* mosquito, bite people and spread the infection. Although other *Aedes* species can act as bearing, their importance is far inferior to that of *Aedes aegypti*. When a mosquito bites a person with DENV infection, the virus first multiplies in the mosquito's gut before moving on to subordinate tissues such as the acinus. The hallmarks of dengue infection appear in a roughly logical order, commencing with viremia and moving on to systemic inflammation and hematological changes [7, 8]. The duration between ingesting the virus and passing it on to a noninfected person is known as the extrinsic incubation period (EIP). The EIP takes about 8 to 12 days at an ambient temperature of 25°C to 28°C. A mosquito bite might cause symptoms to appear 4–7 days later in an affected host. The dengue virus can be transmitted to mosquitos by an infected (asymptomatic) host [4]. In brief, DENV initially infects the mammalian host cells at the periphery, following infection of draining lymph nodes. Virus titers rise in the bloodstream and lymphatics, spreading DENV infection to organs such as the liver and the spleen. Viremia is controlled by the host after a few days, when signs of systemic inflammation are observed. Patients present “cytokine storm,” a term used to describe the high levels of circulating proinflammatory cytokines, which is believed to increase endothelial activation and cause the hemorrhagic manifestations of dengue [8].

For a variety of viral diseases, there are several antiviral medications available in the market such as acetaminophen, aspirin, ibuprofen, and naproxen sodium that are categorized according to their site of action and method of preventing viral reproduction in the host cell. The safety, effectiveness, and cytotoxicity of these antiviral drugs have considerable drawbacks and have a significant difficulty or challenge occurs from the development of resistance, which opens the door for viral genome evolution. This draws away focus from synthetic antiretroviral drugs and towards conventional or alternative medicines, frequently thought to have less ramification and pernicious issues. Natural remedies, also known as phytomedicines, offer a number of advantages over synthetic molecules since they are effective against a variety of host receptors, which enable the treatment of alignment at the ground level through a variety of mechanisms with little or no side effects. This manuscript highlights pathophysiology of the DENV virus, scopes, and potential pharmaceutical targets to treat the dengue with natural medicine including herbs.

## 2. Pathogenesis of Dengue

### 2.1. Viral Entry

**Figure 1:**
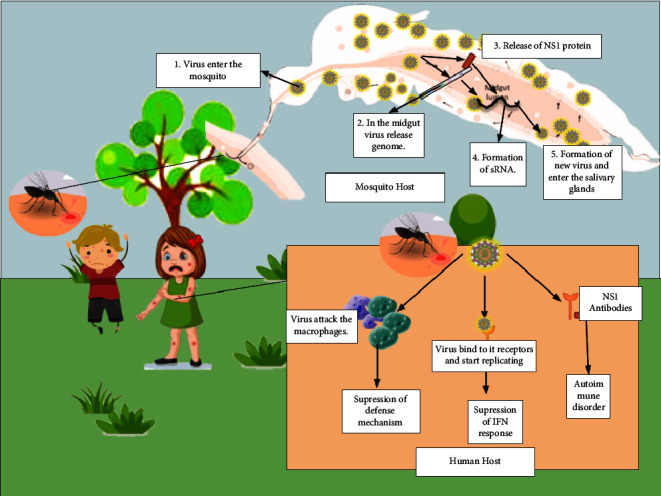
Role of viral and host factor in pathogenesis of dengue fever [22].

**Figure 2:**
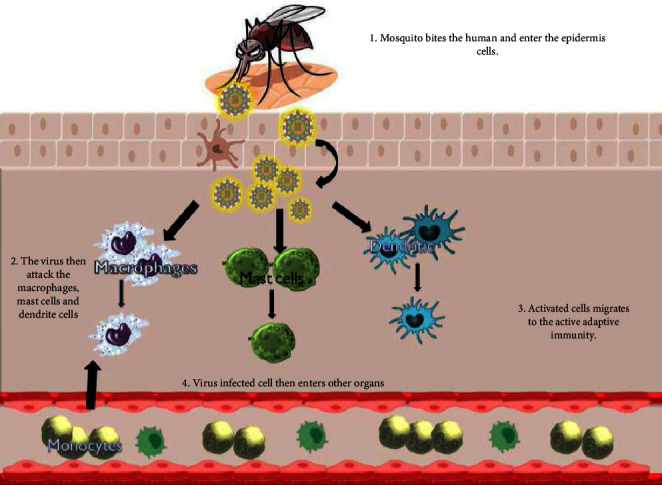
Immunopathogenesis of severe dengue [18].

The method that pathogens utilize to enter their host cells has attracted increased attention in current years. It is thought that keratinocytes and young Langerhans cells in the dermis are the main cells that get infected after a tainted mosquito bite [9]. For last ten years, there have been a lot of research and discussion about the target cell and the DENV section cycle in both vertebrate and invertebrate cells. In any case, the interaction between the sections has not been explained well enough. There has been evidence for a long time that viruses have receptors that change depending on the type of cell they infect [10–17]. DENV is attracted to mononuclear phagocytes, such as leukocyte macrophages, accessory cells, and Langerhans cells in the skin. These cells are thought to be the main targets when DENV is present, even though DENV also affects hepatocytes and endothelial cells [18–20]. These viral receptors connect to carbs in DENV E glycoprotein. In hepatocytes and endothelial cells, DENV also binds to proteins called lectins, vital part-hub unique ICAM-3-getting integrin (L-SIGN), and heparin sulfate-containing proteins [18, 21]. The precise function of the virus and host cell in pathogenesis of dengue fever is illustrated in Figure 1.

van der Schaar et al. [23] showed the beginning stages of DENV in a magnificent way. First, the molecule that causes the infection binds to a cell receptor. In this way, DENV/receptor edifices spread along the surface of the cell until they reach a clathrin-covered pit, where the complex loses its ability to change, as shown in Figure 2. Then, the clathrin-covered pit squeezes into the cytoplasm of the cell, sending the particles to early endosomes that have Rab5 (receptor) on them. Rab-5 and Rab-7 proteins play important roles in the development of endosomes [24]. In general, the early endosome that carries DENV changes into a postendosome by collecting Rab7 over and over again, followed by a gradual lack of Rab5. Lastly, DENV particles can only be found in late endosomes that are Rab7-positive and are prone to pH fermentation. This is needed for DENV to join with the cell layer and spread infection. Cholesterol was found to be a big part of a useful DENV disease in cell and virus films. Cholesterol is thought to be an important part of the viral envelope because treating DENV virions with cholesterol-depleting mixtures, such as methyl-cyclodextrin (MCD), stopped them from spreading. Hence, the need for cholesterol in the virion is a limited part of the four DENV serotypes [25]. Cell cholesterol is usually thought to be related to lipid-pontoons, which act as stages for infection receptors during the passage cycle at the cell surface. On the other hand, DENV replication depends on cholesterol in the intracellular layer to hold the infection's NS (nonstructural) proteins in place [14, 26]. Recent inquisition has revealed a connection between cholesterol and the transmission of viruses as statins impede the transport of DENV E protein in infected cells [27].

### 2.2. Virion Assembly

**Figure 3:**
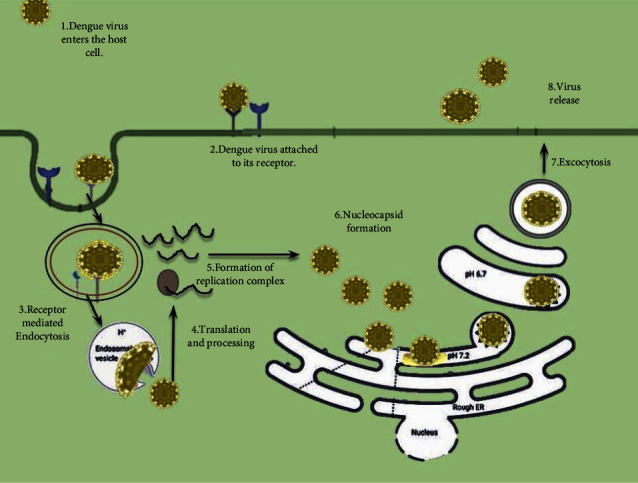
*Flavivirus* life cycle [28].

The nucleocapsid of the dengue virus is made up of viral membrane proteins and envelope glycoproteins E that are buried inside a lipid layer that surrounds genomic RNA and basic capsid C protein (Figure 3). The viral determinant E mediates infection restriction to specific receptors located on the outer layer of Dandy virus-tolerant host cells such as accessory cells (ACs), WBCs, and neural building blocks of life [29]. Only a few of the DENV receptors have been discovered this far, including anticoagulant sulfate, CD14 (cluster of differentiation 14), human laminin receptor, GRP78/BiP, and the adeptly known DC-explicit intercell bond atom three snatching nonintegrin (DC-SIGN) [14–16, 30–33]. After limitation, the infection is taken by the body of receptor-interceded endocytosis. The E protein becomes permanently trimerized as the pH of the endocytosis pathway drops, preventing that capsid from fusing with the cell. When the nucleocapsid is released, the provirus is carried into the protoplasm of infected cells, where it is translated at the unpleasant ER to produce a polyprotein with around 3,400 proteins [34]. Combining cell proteolytic enzymes with the viral nonstructural (NS) 2B-3 proteolytic enzymes results in the co- and post-transformation of this polyprotein into three and seven NS proteins. After interpretation and processing, a polypeptide combination containing the NS5 ribonucleotide bases-subordinate DNA-directed/dependent RNA polymerase, auxiliary NS enzymes, virus (+) genetic material, and perhaps cellular components is collected. Viral RNA copying takes place inside this alleged replication complex (RC), which is linked to infection-induced internal layer formations. Copying starts with a long mixture of genetic (negative) ribonucleic acid, which is used as a blueprint for the creation of (+) ribonucleic acid offspring. The newly blended (−) RNA undergoes base matching with the (positive) ribonucleic acid template during record consummation, resulting in the formation of a long RNA pair known as homologous replication form (RF) [35]. It serves as a replication and hilter-kilter blueprint for the generation of new (+) RNA via a homologous recombination (HR). It can bind to RNA to begin a new interpretation cycle or aggregate to create virions, which are thought to create at the ER (endoplasmic reticulum) when the newly produced (+) RNA ribonucleic acid strands are freed by the RI (replicative intermediate). The golgi bodies are believed to be required for virion maturation and are where the glycosylation of protein molecules as well as the proteolytic cleavage of proteins such as prM (precursor of mature M) into the components pr and M by the golgi resident protease or a similar enzyme occurred [36]. The uncleaved premembrane inhibits E from undergoing a corrosive catalyzed transition into a fusogenic structure during its travel in the secretion route by establishing a prM-E (premembrane and envelop) heterodimeric complex. The pr-fragment is given after cleavage, E creates homodimeric structures, and the experienced viral particles exit the contaminated cell. The *Flavivirus* life cycle is displayed in Figure 3.

## 3. Role of Plant Derived Bioactive Compounds for the Management of DENV

Commonly prescribed symptomatic medications, or so-called current drugs, are highly regarded for their effectiveness and speed of action. On the other side, continuous use might lead to the patient building an immunity to it. The popularity of natural treatments for treating dengue fever, which are significantly less expensive and have no side effects, is a result of the widespread knowledge that these pharmaceuticals, although being too expensive, have certain undesirable side effects. Elucidation of compounds separated from bioactive plants have shown promising results [37]. Organic products from many sources show promise impediment to dengue, either by averting the virus from infiltrating the genome or by pared down the procreate structural and nonstructural proteins. The organic bioactive compounds from plants and a few ocean sources, which inhibit several targets of the dengue virus, are highlighted in this review.

### 3.1. Mimosa Catechu

Mimosa catechu (*Mimosaceae*) is a native plant of South and Southeast Asia, Indian subcontinent, as well as Myanmar, Thailand, and China (Yunnan) [38]. It is a moderate tree with shadowy greyish-brown to dark brown trunk, straight and grey stem, pinnately and alternate leaves, and 5 to 10 cm-long axillary jumps with snowy to light yellow blooms [39]. Major bioactive constituents present in Mimosa catechu are catechin, quercetin, catechol, and catecholamines, and all derivatives of flavanol compounds recovered from the extract of this plant species. The numerous other components present in catechu which demonstrated therapeutic effects are 4-methyl-heptane carboxylic acid methyl ester, methyl laurate, 2-ethyl-3-methylbut-1-ene, tetra decanoic, epicatechin, 4-hydroxybenzoic acid, afzelechin, aromadendrin, kaempferol, and baicalein [40]. Crude extracts from several portions of this plant had been reported to treat diarrhea, diabetes, microbial disease, and in building body immunity. It also has anti-inflammatory, antipyretic, antioxidant, antiulcer, hepatoprotective, and immunomodulatory activity [41]. Acacia's extracts also had antiviral properties against the dengue virus. Reduced peptides found in the DENV outer coating in various DENV types had an influence on the proteins extracted from catechu. According to an analysis, DENV foci formation was persuasively stopped at a half maximal inhibitory dosage of 0.18 g/mL, peptides from Acacia catechu extract shown the highest antidengue efficacy. The viral production was reduced by less than 100 times by administrating 1.25 g/L crude peptide extract [38].

### 3.2. Pawpaw

**Figure 4:**
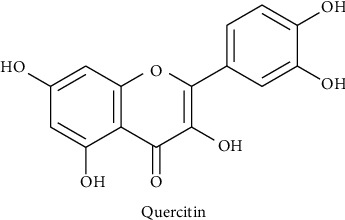
Chemical constituents of pawpaw.

Pawpaw (family: *Caricaceae*) might be a hybrid of two or more *Carica* species endemic to Mexico and Central America [42]. It is a fast-growing, unbranched, tall, annual herb with no branches. The phytochemical profile of immature pawpaw leaves revealed the presence of scopolamine, hydroxybenzene, flavonoids, and amino acids [43, 44]. Quercetin (Figure 4) was one of the *Carica* papaya (CP) bioactive molecules that revealed an antidengue effect. Infections such as dengue could be treated using this method, according to studies at both the transmission and host levels [45]. Within 24 hours, participants in a different trial who routinely consumed 2 tablespoons of pawpaw leaf juice daily noticed a rise in their granulocyte count [46]. Seven days of pawpaw administration to dengue patients with thrombopenia resulted in a significant but variable increase in platelet counts [47]. Similarly in nine clinical studies (6 from India, 1 from Pakistan, 1 from Indonesia, and 1 from Malaysia) matched the criteria for inclusion; CP extract may shorten hospital stays, and the average platelet count from the first to the fifth day of therapy increases [48]. After scrutinized papaya leaf juice for three days following a 24-hour viral infusion elicited a jump in CCL2/MCP-1 levels during the peak of viremia in dengue-infected AG129 mice. Another study revealed that both the methanolic crude and methanolic silver synthesized nanoparticles from C. papaya leaf extracts had high antiviral activities against dengue virus type 2 with IC50 values of 13.09 *μ*g/mL and 09.20 *μ*g/mL, respectively [49].

### 3.3. Punarnava Red

Punarnava red is also called as *Boerhavia diffusa* (family: *Nyctaginaceae*). It is widely dispersed throughout India, the Pacific, and the Southern United States [50]. Ethanolic B. diffusa extract (EBD) and aqueous B. diffusa extract (ABD) were, respectively, appraised for their phytochemical constituents and antioxidant properties. It consists of tannins, total phenols, flavonoids, alkaloids, oxalates, saponins, and phytates [51]. It has been employed as antidiuretic, antibacterial, antiproliferative, antidiabetic, and antifibrinolytic and used as neuro protective [52]. Bharati and Sinha assessed the antidengue cogency of shoots of and Punarnava Linn (10 gm) in patients with dengue infection, and by administering them this extract, the quantity of platelets was raised [53].

### 3.4. *Anacolosa pervilleana*

**Figure 5:**
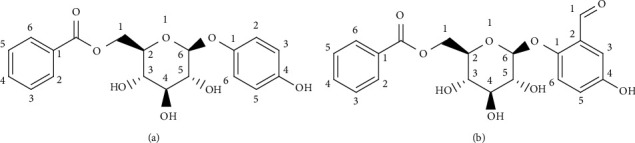
Phytoconstituents of *Anacolosa pervilleana*: (a) *Flacourtia* Side A and (b) *Flacourtia* Side B.

There were 17 species of *Anacolosa pervilleana* (Oleaceae) in Asia, Madagascar, and Africa [54]. In Madagascar, schistosomiasis, syphilis, and general weakness were treated using the young branches, leaves, and hull of *Anacolosa pervilleana* [54]. Diverse bioactive compounds found in *A. pervilleana* were *Flacourtia* Side A, *Flacourtia* Side B (Figure 5), *Flacourtia* Side C, *Flacourtia* Side D, *Flacourtia* Side E, and *Flacourtia* Side F. In the DENV, these bioactives inhibit RNA-dependent RNA polymerase (RDRP) with IC50 value (*μ*M) *Flacourtia* Side A −9.3 ± 2.8 *μ*M, *Flacourtia* Side B −71.1 ± 1.2 *μ*M, *Flacourtia* Side C −23.8 ± 2.7 *μ*M, *Flacourtia* Side D −35.5 ± 3.8 *μ*M, *Flacourtia* Side E −13.4 ± 1.9 *μ*M, and *Flacourtia* Side F −39.8 ± 1.6 *μ*M [54].

### 3.5. Malancha

Malancha is also known as *Alternanthera philoxeroides* belonging to the family: *Amaranthaceae*. It was famous as “alligator weed” in its native places or areas, such as S. Africa, Brazil, Uruguay, and Argentina, and “Malancha” in Bengal. It is an inhabitant of both aquatic and marsh areas, having whitish, papery flowers with hollow, hairy, cylindrical stem, and leaves simple and opposite venation. Out of many constituents found in the plant, coumarin showed antidengue effects [55]. It was investigated for the antiviral activity of four extracts (light petroleum, acetoxy ethane, diethyl ether, and cyclopropyl alcohol). While all of the aforementioned extracts exhibited a noticeable inhibitory impact, *A. philoxeroides*' light petroleum extract had the strongest inhibitory efficacy against DENV [56].

### 3.6. Neem

Neem, scientifically known as *Azadirachta indica*, is a member of the *Meliaceae* family. It was discovered in Africa and Indian subcontinental countries. Therapeutic applications for neem included pain relief, inflammation reduction, fever reduction, infection prevention, and tumor suppression [57]. *In vitro* research demonstrated that *Azadirachta indica* inhibited viral multiplication. The aqueous extract of neem leaves totally suppressed 100–10,000 TCID (tissue culture infectious dose) of the virus at its maximal nontoxic dose of 1.897 mg/ml [58]. Its phytochemistry profile includes Nimbin, 6-deacetylnimbin, desacetylsalannin, and 3-tigloyl azadirachtol. In a computer simulation, nimbin was proven to be efficacious against the protein of all dengue virus types [59]. The verdicts of this study revealed that the triterpenoids nimbin, desacetyl nimbin, and desacetyl salannin, which were widely found in neem plants, have a strong closeness for DENV attachment NS2B-NS3 protein and might be used to generate highly trenchant and propitious medications to treat dengue virus infection [60].

### 3.7. *Bignonia pulchra* Cham

*Arrabidaea pulchra* is notable as *Bignonia pulchra* Cham. belongs to the *Bignoniaceae* family. These species were found in Central America, the Amazon Basin, Argentina, Bolivia, Brazil, and Paraguay. In the treatment of syphilis and other diseases, several species of *Arrabidaea* were utilized [61]. Literature survey revealed that ethanolic extract of *A. pulchra* leaves (EEAPL) was a potential source of the aryl propanoid glycoside derivatives, acetoside and caffeoyl callerianin, respectively, as bioactive molecules which might be the key responsible ingredients for its efficacy against dengue type-2 [62]. The ethanolic extract of *Arrabidaea pulchra* (Cham.) contained caffeine oil calleryanin, verbascoside, and ursolic acid, which were all protective against DENV-2 with EC50 values of 2.8 0.4, 3.4 0.4, and 3.2 0.6 g/mL, and SI values of 20.0, 3.8, and 3.1, respectively.

### 3.8. Green Chiretta

*Andrographis paniculata* (Burm F.) was popularly known as Green chiretta (family: *Acanthaceae*). It grows wild in Asia (namely East Asia, South Asia, and Southeast Asia) and is classified as a green blooming plant. The perennial herb plant lives in hedgerows on flatlands, hill slopes, waste ground, farms, moist environments, shorelines, and along the sides of roadways. *A. paniculata* could also be cultivated in a garden and had erect, luxuriant bifurcated branches [63]. At a minimal nonpoisonous dosage of 15.62 g/mL, andrographolide was examined for its capacity to inhibit DENV-2 [64]. Both the HepG2 and Hela cell lines were used to examine andrographolide's antiviral effect against dengue virus type 2, whereas only the Hela cell line was used to investigate andrographolide's antiviral effect towards DENV serotype 4. *A. paniculata*'*s* EC_50_ were between 21 and 22M for HepG2 and Hela cells, respectively, demonstrating that andrographolide had a potent anti-DENV effect in both cell lines [65].

### 3.9. Turmeric

*Curcuma longa*, another name for turmeric, is a member of the ginger family and is observed in Southeastern Asia, East Asia, and South Asia [66]. It possessed densely branching, fragrant rhizomes ranged in hue from yellow to orange with two rows of alternately vennated leaves. 1,8-cineole, ascorbic acid, eugenol, l-beta-curcumene, volatile oil, diferuloylmethane I (95%), curcumin II (7%), and curcumin III (3%) were all present in it [67]. In many nations, curcumin has been used as a nutraceuticals since ancient times. It has the capability to be used simultaneously as a direct combatant against dengue infections and as a great building block for very effective nonlinear antagonists. Examining the toxicity of *C*. *longa* (0.147 mg/mL) extract in the liver and kidney of DDY (Deutschland, Denken, and Yoken strain) mice, its antiviral effects on DENV-2 were evaluated in preclinical and clinical studies. To assess the antidengue activity, researchers conducted an assay and an MTT test, respectively, in Huh7it-1 cells. To measure viral load, blood samples from infected subjects were obtained 6 and 12 hours after exposure [68]. In plaque tests, curcumin was more efficacious than other drugs at preventing DENV infection, indicating that these drugs target the biochemical reactions required for the virus to mature and reproduce. Anti-DENV activity in Huh7it-1 cells (IC50 17.91 *μ*g/mL) [69, 70]. The NS2B/NS3 protein, which encodes the DENV protease enzyme, was shown to be strongly inhibited in another study. Studying animals in their natural habitat, or *in vivo*, *C. longa* extract at 0.147 mg/mL inhibited DENV-2 replication and shortened the time period during which viremia was present [71].

### 3.10. Licorice

Licorice, as *Glycyrrhiza glabra*, is a member of the *Fabaceae* family and is native to the southern and northern hemisphere [72]. Memory improvement, antipsychotic effects, microbe and cancer protection, free radical scavenger, and healing an ulcer relief were only a few of the therapeutic activities demonstrated by *Glycyrrhiza glabra* root extract. The roots may include a variety of chemical compounds or phytochemicals with a wide range of pharmacological effects [73]. Furthermore, Licorice showed excellent docking characteristics to the protein of dengue [45, 74]. It was shown that glycyrrhizin can inhibit *Flavivirus* procreation at a high nonlethal cytotoxic dose [75]. A study of antiviral phytochemicals identifies three compounds such as Cyanidin 3-O-glucoside chloride, Nigellone, and (R)-Glabridinas potent inhibitors of the dengue virus NS3 protease, which was a key step in the development of an effective vaccine for the mosquito-borne tropical disease dengue fever [76]. In another study, glycyrrhizinic acid showed DENV2 infectivity in Vero E6 cells (IC50 8.1 *μ*M).

### 3.11. Chinese Ginger

Chinese ginger also named as *Boesenbergia rotunda* is a perennial plant belonging to the family of ginger. It might reach a height of fifty centimeters, with a short stem that is replaced by a false stem created by green leaves. Flavonoids such as alpine tin, *Boesenbergia*, cardamonin, and geraniol are examples of the phytochemicals that are found in *B. rotunda* [77, 78]. Antiallergic, antibacterial, antileptospiral, anticancer, antiulcer, antioxidant, antiviral, and antidengue viral actions were among the capabilities of *B. rotunda*. It also has the ability to act as a larvicidal and pupicidal agent and was used to treat hepatic diseases [79]. The bioactive molecules include cyclohexenyl chalcone derivatives, pinostrobin, (−) panduratin A, and 4- hydroxy panduratin from the Boesenbergia rotunda plant exhibited potent competitive inhibitory effects against the NS3 protease of the dengue 2 viruses with IC50 values as 90.48, 235.86, 242.76, 273.10, and 286.90 [80].

### 3.12. Nutmeg

The *Myristicaceae* category contained nutmeg, also known as *Myristica fatua*. It is a fragrant tree that grows in Bangladesh, India, and Indonesia and produces yellow organic fruits that resemble apricots or peaches. It was once used in herbal medicine as an antioxidant, analgesic, amenorrheal, aphrodisiac, and digestive specialist [81]. The results of the study indicated that nutmeg methanolic extract magnificently reduced DENV infection up to 21.61 percent devoid of the cytostatic effect. Furthermore, the antidengue effect against DENV with EC50 was 25.33 *μ*g ml^−1^ [82].

### 3.13. *Webera corymbosa*

*Chomelia asiatica*, also recognized as *Webera corymbose* (family: *Rubiaceae*), is typically grown in India. In the past, *Webera corymbosa* leaf, barks, and underlying structures were employed in ancient Ayurvedic medicine to cure a wide range of illnesses [83]. DENV-infected C6/C36 cells that were treated with acetone extracts of *Webera corymbosa* reported a CC50 value of 34.35 percent at a concentration of 500 micrograms per milliliter [84].

### 3.14. Mangosteen

The mangosteen, known as *Garcinia mangostana* (MG), belongs to the family *Guttiferae*, also recognized as “the sovereign of natural goods.” This tropical evergreen tree is found in India, Sri Lanka, Myanmar, Malaysia, the Philippines, and Thailand [50, 85]. It contains significant phytoconstituents mangosteen, acetoside, and various potent xanthene derivatives [86]. The IC50 values were 5.47 *μ*M 24 h treatments, inhibiting virus replication. The infection rates of four different dengue virus types were lowered by 40–60 percent when DENV-infected cells were treated with MG (20 M). It diminishes the production of dengue virus type 2, 3 by 100 folds, whereas in dengue 1, 3 type, additionally, it could drastically reduce the body's production of mediators and inflammatory markers. The effects of -MG were superior to those of the anti-inflammatory drug and the antiretroviral ribavirin [87].

### 3.15. *Euphorbia hirta* (Asthma-Plant)

Asthma plant (family: *Euphorbiaceae*), develops in open fields in India, Ceylon, British Malaya, Java, and Socialist Republic of Vietnam, is well known for its folklore medicinal applications [88]. Asthma plant is a small, hairy, annual herb with branches that reach a height of 60 cm, is scarlet or purple, and generates large amounts of latex. The herb was reportedly used as a spasmolytic, alpha-glucosidase inhibitor, anti-inflammatory, antitumor, and antidengue agent [89]. Patients were able to recover more rapidly because to Euphorbia hirta's promotion of platelet formation, which decreased further bleeding. The natives of the Philippines also took Euphorbia hirta-made capsules to treat dengue. Euphorbia inhibited DENV-1, DENV-2, DENV-3, and DENV4 (IC50 = 33.84, 33.55, 58.35, and 119 *μ*g/mL), respectively [88].

### 3.16. Hauili

Hauili, also known as *Ficus septica* (family: *Moraceae*), is a fig that is indigenous to Malasia, Australia Terra Australis, South China, Republic of China, and Land of Seven Sisters [90]. The majority of the world's tropical wet forests contain more than 750 varieties of woody plants of the genus *Ficus*, described by Morceau. A deciduous shrub or small tree with aerial roots that resemble a bush, *Ficus deltoidea* frequently starts off as an epiphyte. With branches that were around 15–22 feet high and 3–10 feet wide, it frequently reached a height of 22 feet. The bark and trunk were commonly grey and thin, respectively [91]. In conventional medicine, they had been used as a carminative, stomachic, microbicide, in postural hypotension, and as an antidysentery medication. Gardens were planted with a wide variety of plants for decoration and shade. [92]. WS1 human foetal skins typical fibroblasts cells, HepG2 human hepatoma, Huh7.5 human hematoma, and A549 human lung epithelial carcinoma cells were only a few of the cell types that Hauili in the Philippines leaf methanol shielded from DENV contamination [53, 93]. The immunofluorescence experiment demonstrated that the methanol extracts of *Ficus septica's* fruit, heartwood, leaves, and stem exhibited a potential anti-DENV-1 and DENV-2 impact. DENV-1 (IC50 = 17.44.6 g/ml) and DENV2 (IC50 = 15.82.5 g/ml) were strongly suppressed by FS-(L)-M [94].

### 3.17. Red Fire

*Hemigraphis reptans*, often known as red flame (Family: Acanthaceae), are native Malaysian green plants and are once used to treat wounds, inflammation, diabetes mellitus, iron-deficiency anemia, heavy menstruation, and gallstones [95]. Evidence suggested that *Hemigraphis* leaf extracts assessed positive results against DENV-2 and NS2B-NS3 protease with IC50 values of 100 g ml for the management of dengue fever [96].

### 3.18. *Ocimum sanctum*

*Ocimum tenuiflorum*, also identified as *Ocimum sanctum* (family: *Lamiaceae*), believed to have hepatoprotective, calming, and antiviral properties [97]. It prevented hiccups, fever, and ulcers. *Ocimum sanctum* L. (Labiatae) is a tiny, highly perfumed annual plant that grows to a height of eighteen feet and is often called holy basil, Tulsi. *Ocimum sanctum* L. contains vitamin C and A; minerals such as calcium, zinc, and iron; as well as chlorophyll and other flavonoids [98]. It had been shown to have qualities that were antidiabetic, bruise-healing, tocopherol, actinotherapy, antiangiogenic, oral contraceptives, anti-inflammatory, antibacterial, antistress, and antitumor [99]. In addition, *O. sanctum* methanolic extract displayed antiviral activity against DENV-1 by inhibiting CPE development and viral replication. The methanol extract of *A. paniculata* at its MNTD only inhibits CPE formation, not viral replication, which is how the antidengue-1 cytotoxic activity is achieved. The effectiveness of methanolic extracts of *Andrographis paniculata* and *Ocimum sanctum* against DENV1 was examined using HepG2 cells. Prior to the antiviral experiment, the maximum nontoxic dose (MNTD) of the extract and the median tissue culture infective dose (TCID50) of DENV-1 against HepG2 cells were determined. The antiviral activity was assessed by measuring the degree of inhibition based on cytopathic effects (CPE), cell survival using the MTT test, and plaque inhibition assay [100].

### 3.19. *Magnolia berry*

Typically growing in northeastern China, magnolia-vine also known as *Magnolia berry* (family: *Schisandraceae*) is a flora whose natural products had been used to treat alimentary tract and digestive tract problems, respiratory difficulties, blood-vascular issues, body exhaustion, excessive sweating, and sleeplessness to show nonsteroidal anti-inflammatory drugs, antiviral, and neuroprotective characteristics [101]. Three days of schisandrin derivatives (active constituent) treatment were given to Huh-7 cells that had DENV infection. Using RT-PCR and western blotting, respectively, DENV RNA and protein levels were assessed; the amount of NS2B represented the level of DENV protein production. On comparing treated cells with crude cells, schisandrin A effectively reduced DENV RNA and protein levels. The supression of DENV RNA and protein content in cells treated with schisandrin B and C is lesser than cells treated with schisandrin A. EC50 values for schizandrol A, schizandrol B, and schizandrol C were discovered to be 28.1 0.42 M, 34.0 0.95 M, and 42.6 3.48 M, respectively. As the cells were treated to either schisandrin analogue at possible antibacterial dosages, no obvious cytotoxicity was seen. Consequently, schisandrin A was chosen as a promising inhibitor [102].

### 3.20. *Governor's plum*

*Governor's plum* or *Flacoutia ramontchi* (family: *Salicaceae*) is a conifer tree native to Madagasikara and Indochinese Peninsula [103]. Potential actions include inflammation inhibiting, antimalarial, antiviral, and snake venom phosphodiesterase inhibiting. Active constituents of *Flacourtia ramontchi* embraces daucosterol; sitosterol; phenolic glycoside flavonoids such as robigenin, three rutinoside, and quercetin; flacosides including flacoside A, B, and C polioithrysoside; and salirepin [103, 104]. In another study, Betulinic acid 3-caffeate, ramontchi, A, and E, as well as scolochinenoside D, severely hindered RNA polymerase action in the dengue virus RNA polymerase experiment. However, in the experiment, betulinic acid 3-caffeate and flacourtia sides A and E along with scolochinenoside D dramatically reduced RNA polymerase activity (IC50 = 0.85 *μ*M, 0.1 *μ*M, 1 *μ*M, and 5 *μ*M, respectively). For both tests, *Flacourtia ramontchi's* stem bark extract was shown to be the most effective. This was the first-timebioassay-guided isolation, yielded six novel phenolic glycosides, together with poliothrysoside, flacourtosides (A–F), xylosmin, scolochinenoside D, betulinic acid 3-caffeate, and itoside H [104].

### 3.21. *Cryptocarya chartacea*

There were a variety of secondary compounds found in *Cryptocarya chartacea* (Family: Lauraceae), flavonoids, such as pyrones, that had demonstrated biological actions such as antiviral and cytostatic activity [105]. Selecting new caledonian plants with considerable dengue virus RDRP (RNA dependent RNA polymerase) inhibitory activity was made possible by an *in vitro* screening of the plants. chartaceones A–F, together with pinocembrin were isolated from *Cryptocarya chartacea* during a chemical examination of the plant. Racemic mixtures were used to extract them, and one- and two-dimensional NMR spectroscopy was used to describe them. By comparing their experimental and predicted spectra with chiral HPLC, the four diastereomers of chartaceone A were identified and their absolute configurations were identified. The strongest NS5 RDRP inhibitors have IC50 values in the range from 1.8 to 4.2 M and are dialkylated flavanones such as chartaceones CF. This family of non-nucleoside DENV RDRP inhibitors was called Chartaceones [105].

### 3.22. Chinese Lizard Tail

*Chinese lizard tail*, also known as fish mint (family: *Saururaceae*), primarily found in Eastern Asia, mainly China, is a plant that experienced seasonal regrowth and springtime regrowth. It had counteracted the effects of leukemia having anticarcinogenic, anti-inflammatory, and anti-anaphylaxis properties [106]. To minimize side effects of clinically used medications, fish mint, an edible vegetable in China, was notified to be an effective supplement. Phytochemicals found in *H. cordata* include flavonoids, alkaloids, fatty acids, sterols, polyphenolic acids, sterol esters, and quercetin, out of which quercetin showed antidengue effects along with antiproliferative, antioxidant, antidiabetic, antihypertension, and antimutagenic potential [107]. According to the study's findings, pre- and postbreeding with *H. cordata* extract dramatically decreased the synthesis of internal DEN-2 RNA, which related to a decrease in zika protein production. At an effective dose (EC50) of 0.8 mg/mL, the extract was bound to DEN-2 and dramatically reduced intracellular RNA synthesis when used in the straight block mode of action. Furthermore, the aqueous extract of *Houttuynia cordata's* showed inhibitory effects on dengue virus and cells with dengue infection in another study [108]. In addition, quercetin or isoquercetin, two significant water extractable flavonoids, were present in *H. cordata* hot water extracts at 10 M, they inhibited HSV-2 infection by blocking NF-B activation [109, 110].

### 3.23. Baikal skullcap

Baikal was a type of flower plant of the *Lamiaceae* family, native to both Russia and East Asia. *Scutellaria baicalensis* had roughly 360 different species worldwide. Scutevulin and carthamidin and baicalin were included in the list of ingredients for their medicinal characteristics, as well as kaempferol 3-O-D-glucoside for its antioxidant capabilities [111, 112]. Baicalin was demonstrated to target nonstructural viral proteins of DENV-2 internal replicon [113]. Baicalin, a bioactive metabolite from Baikal, indicated potent antidengue activity [114, 115]. Delving further revealed that the *S. Baicalensis* extract particularly earmarked certain DENV infection and proliferation phases. Studies also showed that *S. baicalensis* extract had a strong direct virucidal activity that could neutralize exogenous DENVs that were circulating in patients with viremic disease. This activity served as a crucial criterion for the creation of antidengue drugs. The presence of baicalein, a flavonoid renowned for its ability to inhibit the reproduction of the dengue virus, may be one of the naturally occurring antiviral components, according to findings that also highlighted the potential of *S. baicalensis* aqueous extract for use against the dengue virus [116].

### 3.24. *Distictella elongata*

*Distictella elongata* (family: *Bignoniaceae*) was originated in Minas Gerais, Brazil. It has been claimed to have antimicrobial effects versus DENV-2 in addition to its antioxidant and anticancer activities. It might be useful as a source of pectolinarin due to its previously documented antiviral, anti-inflammatory, and collagen-inducing activities. *Distictella elongata* fruit ethyl alcohol extracts were used to extract acacetin-7-O-rutinoside and pectolinarin, both of which had anti-DENV-2 activity (EC50 = 9.8 *μ*g/ml) [117, 118].

### 3.25. Brown Algae

Hippo Thesaurus established as “Mojuka” or *Cladosiphon okamuranus* was consumed in Nippon. Active principle, fucoidan, provided antiulcer and gastric mucosa protection, according to *in vitro* tests [119]. The exterior proteoglycans (EGP) of DENV-2 may interact with sulfated polysaccharides (fucoidans) enabling them to entirely adhere to the virus, act against DENV -2, -3, -4 with IC50 = 4.7, 500, and 365 *μ*g ml^−1^ [120].

### 3.26. Red Rea Weed

*Gymnogongrus griffithsiae*, a polysiphonia, a native to Brazil, belongs to the *Phyllophoraceae* family contains unrefined polysiphonia extract sulfated with galactan. Two identical sulfated starches from red seaweeds, carrageenan, and rhamnan were tested for their antiviral activity against the four dengue virus subtypes (DENV) in various host cell types. Red seaweed has been shown to be resistant to Herpes simplex types I and II [121]. Red seaweed revealed a selective antagonist of dengue virus type-2 multiplication in Vero cells but had a less potent antiviral effect against dengue virus types 3 and 4 [122].

### 3.27. Moreton Bay Chestnut

Black bean or Moreton Bay chestnut, *Castanospermum australe*, is a native to Australia and could also be noticed in Asia. The seeds of this plant, which contained active constituent castanospermine, were poisonous to horses and could make people sick and after being carefully converted to fine powder, they could be consumed [123]. DENV-1, 2, 3, and 4 subtype cellular exudation and dissemination were reported to be inhibited by castanospermine both *in vitro* and *in vivo* [124].

### 3.28. *Cissampelos pareira*

In India, traditional uses for velvet leaf (family: *Menispermaceae*) included purification of blood and pain relief. Cissampelo flavone that worked well against all four types of dengue virus. The root of this plant could help wounds heal and was also known to relieve pain and ease joint pain. *C. pareira* inhibited DENV-2*in vivo* and prevented mouse mortality with IC50 = 85.7 *μ*M [125, 126].

A summary of all the plant products including part of the plant used, targets, and their uses as antidengue agents and their IC50/EC50 values is provided in Tables 1 and 2, respectively.

## 4. Conclusion and Future Perspectives

Notwithstanding tremendous progress, attempts being made to develop dengue vaccines or treatment regimens have not yet shown satisfactory results. Many research organizations are working towards the development of antivirals to treat DENV infection, which will indeed assist in the creation of this vital resource for the treatment of severe sickness. Natural products are a great source of biodiversity for the development of novel antivirals, with new structure-activity interactions, and efficient preventative and therapeutic approaches to viral diseases. It has been further concluded that plants such as *Governor's plum*, *Cryptocarya chartacea, Magnolia berry,* and *Chinese ginger* revealed many active pharmaceutical constituents against DENV and could be a promising target for drug discovery. In addition, more investigation is required to pinpoint the most suitable stages to stop the spread of virus infection and by concentrating on each stage of the viral life cycle, newfangled molecules may be developed to stop the infection of host cells, viral maturation, viral RNA synthesis, and viral particle spread. To aid in the development of effective antiviral treatments, further exploration in defining the underlying mechanisms, describing the bioactive ingredients, as well as assessing the efficacy and potential application *in vivo* is encouraged since many studies in this field are still in the preliminary stages. A multitargeted therapy may also help to lower the likelihood of creating drug resistant viruses. Subsequent research should also investigate the possibilities of combination therapies with other natural agents or with conventional pharmaceuticals. Hence, the development of antiviral medications will benefit greatly from natural items in the future.

## Figures and Tables

**Table 1:** Plants used in the treatment of DENV and other ailments.

Name of plant	Family	Targeted DENV serotype	Uses	Parts of plant used	Reference
*Acacia catechu*	Mimosaceae	DENV-1,-2,-3,-4	Anti-inflammatory and antidiarrhea, and antidengue	Dried fruit (powder form)	[38]
*Carica papaya*	Caricaceae	DENV-2	Treats dengue, jaundice, obesity, fever, and asthma	Leaves	[49]
*Boerhavia diffusia*	Nyctaginaceae	DENV serotypes not mentioned	Treats gastrointestinal, antidengue hepatic, and gynecological illnesses	Whole plant	[53]
*Arrabidaea pulchra*	Bignoniaceae	DENV-2	Anti-inflammatory, astringent and antisyphilitic agents, and antidengue	Leaves	[61]
*Curcuma longa*	Zingiberaceae	DENV-2	Anti-inflammatory, antidengue, antioxidant, antidiabetic, and anticancer	Whole plant	[68]
*Glycyrrhiza glabra*	Leguminosae	DENV protease	Antibacterial, anti-oxidant, anti-inflammatory, antidengue, antihyperglycemic, and antiviral	Whole plant	[74]
*Garcinia mangostana*	Guttiferae	DENV-1, -2, -3, -4	For abdominal pain, dysentery, wound infection, ulcer, diarrhea, and antidengue	Pericarp	[127]
*Ficus septica*	Moraceae	DENV-1 and DENV-2	Laxative and antidengue	Fruit, heartwood, leaves, and stem	[93]
*Anacolosa pervilleana*	Olacaceae	DENV 5 NS5 polymerase	Treats schistosomiasis and antidengue	Leaves	[95]
*Scutellaria baicalensis*	Lamiaceae	DENV-2	Antidengue	Roots	[114]
*Distictella elongata*	Bignoniaceae	DENV-2	Antidengue	Leaves, fruits, and stems	[118]
*Cladosiphon okamuranus*	Chordariaceae	DENV -2, −3, −4	Antidengue	Pickled seaweed	[120]
*Gymnogongrus griffithsiae*	Phyllophoraceae	DENV-2, DENV-3 and DENV-4, and inactive against DENV-1	Antidengue	Whole plant	[122]
*Castanospermum australe*	Fabaceae	DENV-1, -2, -3, -4	Antidengue	Seeds	[124]
*Cissampelos pareira*	Menispermaceae	DENV-1, -2, -3, -4	Antidengue	Aerial parts	[126]
*Alternanthera philoxeroides*	Amaranthaceae	DENV-2 NS5 polymerase	Antidengue	Purchased andrographolide	[128]
*Azadirachta indica*	Meliaceae	DENV-2	Anti-inflammatory, antiarthritic, antipyretic, antidengue, antifungal, antibacterial, and antitumor agent	Leaves	[129]
*Andrographis paniculata*	Acanthaceae	DENV-2 and DENV-4	Treats sore throat, flu and respiratory infection, anti-cancer, anti-inflammatory, antibacterial, antidiabetic, and antiviral activities	Whole plant	[128]
*Boesenbergia rotunda*	Zingiberaceae	DENV-2	Treats inflammation, fever, gout, flatulence, stomachache, and dyspepsia	Fingerroot	[130]
*Myristica fatua*	Myristicaceae	DENV-2	Anti-oxidant, analgesics, amenorrhea, aphrodisiacs, and digestive agents	Whole plant	[131]
*Tarenna asiatica*	Rubiaceae	DENV serotypes	Antidengue	Leaves	[84]
*Euphorbia hirta*	Euphorbiaceae	DENV serotypes	Antidengue	Whole plant	[132]
*Hemigraphis reptans*	Acanthaceae	DENV serotypes	Treats excessive menstruation, inflammation, diabetes, anamia, and antidengue	Whole plant	—
*Ocimum sanctum*	Lamiaceae	DENV-1	Preventing cough, fever, ulcer hepatoprotective, anti-inflammatory, and antiviral properties	Whole aerial body	[133]
*Schisandra chinensis*	Schisandraceae	DENV-1,-2,-3,-4	Disorders of the gastrointestinal, antidengue, respiratory failure, cardiovascular diseases, body fatigue, and excessive sweating	Whole plant	[134]
*Houttuynia cordata*	Saururaceae	DENV-2		Whole plant	[135]

**Table 2:** Plants showing antidengue activity with IC50-EC50 values.

S. no	Plant	Description	EC50/IC_50_	Reference
1	*Acacia catechu*	Antidengue activity of catechu peptides on DENV foci	0.18 *μ*g/ml	[38]
2	*Carica papaya*	Dengue virus type 2 is inhibited by methanolic crude and methanolic silver manufactured nanoparticles from *C. papaya* leaf extracts	13.09 *μ*g/ml 09.20 g/ml	[49]
3	*Anacolosa pervilleana*	Bioactive compounds inhibited RNA-dependent RNA polymerase (RDRP)	IC50 value (*μ*M) flacourtoside A 9.3 ± 2.8, flacourtoside B −71.1 ± 1.2, flacourtoside C −23.8 ± 2.7, flacourtoside D −35.5 ± 3.8, flacourtoside E −13.4 ± 1.9, and flacourtoside F −39.8 ± 1.6	[54]
4	*Arrabidaea pulchra*	The caffeine oil calleryanin, verbascoside, and ursolic acid found in the ethanol extract of Arrabidaea pulchra pretended against DENV-2	EC50 = 2.8 ± 0.4, 3.4 ± 0.4, and 3.2 ± 0.6 g/ml	[62]
5	*Andrographis paniculata *	Andrographolide exhibited a strong anti-DENV impact in HepG2 and hela cell lines	EC50 = 21 and 22 *μ*M	[65]
6	*Curcuma longa*	Anti-DENV potential against Huh7it-1 cell	IC50 = 17.91 = *μ*g/ml	[69]
7	*Glycyrrhiza glabra*	Glycyrrhizic acid demonstrated anti-DENV2 infectivity in Vero E6 lines	IC50 = 8.1 *μ*M	[76]
8	*Boesenbergia rotunda*	Chalcone, cardamonin, Alpinetin, pinocembrin, and pinostrobin, acted against dengue virus type 2 (DENV 2) NS3 protease	IC50 (*μ*M) pinostrobin −90.48, cardamonin −235.86, alpinetin −242.76, pinocembrin −273.10, and chalcone −273.10	[80]
9	*Myristica fatua*	DENV	EC50 = 25.33 *μ*g/ml	[82]
10	*Garcinia mangostana*	DENV	IC50 = 5.47 *μ*M	[87]
11	*Ficus septica*	DENV-1 DENV2	DENV-1 (IC50 = 17.44.6 g/ml) and DENV2 (IC50 = 15.82.5 g/ml)	[94]
12	*Scutellaria baicalensis*	DENV-2	(IC50 = 1.55 *μ*g/ml	[116]
13	*Cryptocarya chartacea*	DENV NS5 and RNA-dependent RNA polymerase	IC50 = 1.8 to 4.2 *μ*M	[105]
14	*Flacoutia ramontchi*	Betulinic acid 3*ß*-caffeate, flacourtia aSides A and *E*, and scolochinenoside D significantly decreased RNA polymerase activity by inhibiting DENV replication	IC50 = betulinic acid 3*ß*-caffeate 0.85 ± 0.1 *μ*M, flacourtia Side A = 0.1 *μ*M, flacourtia Sides *E* = 1 *μ*M and scolochinenoside D = 5 *μ*M, respectively	[104]
15	*Distictella elongata*	DENV-2	EC50 = 9.8 *μ*g/ml	[117]
16	*Cladosiphon okamuranus*	DENV -2, −3, −4	IC50 = 4.7, 500, 365 *μ*g/ml	[120]
17	*Gymnogongrus griffithsiae*	IC50 = 0.9 g/mL, specifically suppressed DENV-2 replication	IC50 = 0.9 *μ*g/ml	[122]
18	*Castanospermum australe*	Only inhibited DENV-2 *in vivo* and prevented mouse mortality	(IC50 = 85.7 *μ*M	[124]
19	*Cissampelos pareira*	DENV-1, -2, -3, -4	IC 50 = 2 *μ*g/ml	[125, 126]
20	*Myristica Fatua*	Myristica fatua's methanolic extract inhibited the DENV-2 NGC	EC50 = 25.33 *μ*g/ml	[82]
21	*Euphorbia hirta*	DENV-1, DENV-2, DENV-3, and DENV4	IC50 = 33.84, 33.55, 58.35 and 119 *μ*g/ml	[88]
22	*Schisandra chinensis*	DENV 1, 2, 3, 4	EC50 = 28.1 ± 0.42 *μ*M	[102]
23	*Houttuynia cordata*	Inhibited intracellular viral RNA replication	EC50 = 0.8 g/ml	[109, 110]
24	*Magnolia berry*	Schizoandrol A, schizoandrol B, and schizoandrol C screened against DENV	EC50 values of schizoandrol *A* = 28.1 ± 0.42 M, schizoandrol B = 34.0 ± 0.95 M, and schizoandrol C = 42.6 ± 3.48 M	[102]

## Data Availability

The data that support the findings of the study are available from the corresponding authors upon request.

## References

[1] Aguiar M., Anam V., Blyuss K. B. (2022). Mathematical models for dengue fever epidemiology: a 10 year systematic review. *Physics of Life Reviews*.

[2] Wang W. H., Urbina A. N., Chang M. R. (2020). Dengue hemorrhagic fever – a systemic literature review of current perspectives on pathogenesis, prevention and control. *Journal of Microbiology, Immunology, and Infection*.

[3] Jena N., Bal C., Sharon A. (2019). Plant and marine products: a promising hope in the search of therapeutics against dengue. *Discovery and Development of Therapeutics from Natural Products against Neglected Tropical Diseases*.

[4] Chavda V. P., Kumar A., Banerjee R., Das N. (2022). Ayurvedic and other herbal remedies for dengue: an update. *Clinical Complementary Medicine and Pharmacology*.

[5] Chen H. R., Lai Y. C., Yeh T. M. (2018). Dengue virus non-structural protein 1: a pathogenic factor, therapeutic target, and vaccine candidate. *Journal of Biomedical Science*.

[6] San Martín J., Brathwaite O., Zambrano B. (2010). The epidemiology of dengue in the americas over the last three decades: a worrisome reality. *American Journal of Tropical Medicine and Hygiene*.

[7] Guzman M. G., Kouri G. (2002). Dengue: an update. *The Lancet Infectious Diseases*.

[8] Marques R. E., Guabiraba R., Cisalpino D., Teixeira M. M., Souza D. G. (2014). Dengue. *Colloquium Series on Integrated Systems Physiology: From Molecule to Function*.

[9] Limon-Flores A. Y., Perez-Tapia M., Estrada-Garcia I. (2005). Dengue virus inoculation to human skin explants: an effective ap-proach to assess in situ the early infection and the effects on cutaneous dendritic cells. *International Journal of Experimental Pathology*.

[10] Bielefeldt-Ohmann H., Meyer M., Fitzpatrick D. R., Mackenzie J. S. (2001). Dengue virus binding to human leukocyte cell lines: receptor usage differs between cell types and virus strains. *Virus Research*.

[11] Jindadamrongwech S., Smith D. R. (2004). Virus Overlay Protein Binding Assay (VOPBA) re-veals serotype specific heterogeneity of dengue virus binding proteins on HepG2 human liver cells. *Intervirology*.

[12] Martínez Barragán J. d., del Angel R. M. (2001). Identification of a putative coreceptor on Vero cells that participates in dengue 4 virus infection. *Journal of Virology*.

[13] Moreno-Altamirano M. M. B., Sanchez-Garcia F. J., Munoz M. L. (2002). Non Fc receptor-mediated infection of human macrophages by dengue virus serotype 2. *Journal of General Virology*.

[14] Navarro-Sanchez E., Altmeyer R., Amara A. (2003). Dendritic-cell-specificICAM3-grabbing non-integrin is essential for the productive infection of human dendritic cells by mosquito-cell-derived dengue viruses. *EMBO Reports*.

[15] Reyes-Del Valle J., Chávez Salinas S., Medina F., del Angel R. M. (2005). Heat shock protein 90 and heat shock protein 70 are components of dengue virus receptor complex in human cells. *Journal of Virology*.

[16] Thepparit C., Smith D. R. (2004). Serotype-specific entry of dengue virus into liver cells: identifica-tion of the 37-kilodalton/67-kilodalton high-affinity laminin receptor as a dengue virus serotype 1 receptor. *Journal of Virology*.

[17] Wei H. Y., Jiang L. F., Fang D. Y., Guo H. Y. (2003). Dengue virus type 2 infects human endothelial cells through binding of the viral envelope glycoprotein to cell surface polypeptides. *Journal of General Virology*.

[18] Jessie K., Fong M., Devi S., Lam S., Wong K. (2004). Localization of dengue virus in naturally infected human tissues, by immunohis-tochemistry and in situ hybridization. *The Journal of Infectious Diseases*.

[19] Couvelard A., Marianneau P., Bedel C. (1999). Report of a fatal case of dengue infection with hepatitis: demonstration of dengue antigens in hepatocytes and liver apoptosis. *Human Pathology*.

[20] Tassaneetrithep B., Burgess T. H., Granelli-Piperno A. (2003). DC-SIGN (CD209) mediates dengue virus infection of human dendritic cells. *Journal of Experimental Medicine*.

[21] Miller J. L., deWet B. J. M., Martinez-Pomares L. (2008). The mannose receptor mediates dengue virus infection of macrophages. *PLoS Pathogens*.

[22] Bhatt P., Sabeena S. P., Varma M., Arunkumar G. K. (2020). Current understanding of the pathogenesis of dengue virus infection. *Current Microbiology*.

[23] van der Schaar H. M., Rust M. J., Chen C. (2008). Dissecting the cell entry pathway of dengue virus by single-particle tracking in living cells. *PLoS Pathogens*.

[24] Rink J., Ghigo E., Kalaidzidis Y., Zerial M. (2005). Rab conversion as a mechanism of progression from early to late endosomes. *Cell*.

[25] King C. A., Marshall J. S., Alshurafa H., Anderson R. (2000). Release of vasoactive cytokines by antibody-enhanced dengue virus infection of a human mast cell/basophil line. *Journal of Virology*.

[26] Lee C. J., Lin H. R., Liao C. L., Lin Y. L. (2008). Cholesterol effectively blocks entry of flavivirus. *Journal of Virology*.

[27] Martinez-Gutierrez M., Castellanos J. E., Gallego-Gomez J. C. (2011). Statins reduce dengue virus production via decreased virion assembly. *Intervirology*.

[28] Suthar M. S., Diamond M. S., Gale Jr M., West M. (2013). West Nile virus infection and immunity. *Nature Reviews Microbiology*.

[29] Anderson R. (2003). Manipulation of cell surface macromolecules by flaviviruses. *Advances in Virus Research*.

[30] Chen Y. C., Wang S. Y., King C. C. (1999). Bacterial lipopolysaccharide inhibits Dengue virus infection of primary human monocytes/macrophages by blockade of virus entry via a CD14-dependent mechanism. *Journal of Virology*.

[31] Jindadamrongwech S., Thepparit C., Smith D. R. (2004). Identification of GRP 78 (BiP) as a liver cell expressed receptor element for Dengue virus serotype 2. *Archives of Virology*.

[32] Lozach P. Y., Burleigh L., Staropoli I. (2005). Dendritic cell-specific intercellular adhesion molecule 3-grabbing non-integrin (DC-SIGN)-mediated enhancement of Dengue virus infection is independent of DC-SIGN internalization signals. *Journal of Biological Chemistry*.

[33] Salas-Benito J., Valle J. R. D., Ceballos-Olvera I., del Angel R. M., Salas-Benito M., Mosso C. (2007). Evidence that the 45-kD glycoprotein, part of a putative Dengue virus receptor complex in the mosquito cell line C6/36, is a heat-shock related protein. *The American Journal of Tropical Medicine and Hygiene*.

[34] Bartenschlager R., Miller S. (2008). Molecular Aspects of Dengue Virus Replication. *Future Microbiology*.

[35] Lescar J., Soh S., Lee L. T., Vasudevan S. G., Kang C., Lim S. P. (2018). The dengue virus replication complex: from RNA replication to protein-protein interactions to evasion of innate immunity. *Dengue and Zika: Control and Antiviral Treatment Strategies*.

[36] Ferrari M., Zevini A., Palermo E. (2020). Dengue virus targets Nrf2 for NS2B3-mediated degradation leading to enhanced oxidative stress and viral replication. *Journal of Virology*.

[37] Martinez J. P., Sasse F., Brönstrup M., Diez J., Meyerhans A. (2015). Antiviral drug discovery: broad-spectrum drugs from nature. *Natural Product Reports*.

[38] Panya A., Yongpitakwattana P., Budchart P. (2019). Novel bioactive peptides demonstrating anti-dengue virus activity iso-lated from the Asian medicinal plant Acacia catechu. *Chemical Biology &amp;amp; Drug Design*.

[39] Adhikari B., Aryal B., Bhattarai B. R. (2021). A comprehensive review on the chemical composition and pharmacological activities of Acacia catechu (lf) willd. *Journal of Chemistry*.

[40] Wang L., Shen X., Mi L. (2019). Simultaneous determinations of four major bioactive components in Acacia catechu (Lf) Willd and Scutellaria baicalensis Georgi extracts by LC–MS/MS: application to its herb–herb interactions based on pharmacokinetic, tissue distribution and excretion studies in rats. *Phytomedicine*.

[41] Stohs S. J., Bagchi D. (2015). Antioxidant, anti‐inflammatory, and chemoprotective properties of Acacia catechu heartwood extracts. *Phytotherapy Research*.

[42] Kaur M., Talniya N. C., Sahrawat S., Kumar A., Stashenko E. E. (2019). Ethnomedicinal uses, phytochemistry and pharmacology ofcarica papaya plant: a compendious review. *Mini-Reviews in Organic Chemistry*.

[43] Akhila S., Vijayalakshmi N. G. (2015). Phytochemical studies on Carica papaya leaf juice. *International Journal of Pharmaceutical Sciences and Research*.

[44] Ayoola P. B., Adeyeye A. (2010). Phytochemical and nutrient evaluation of Carica papaya (pawpaw) leaves. *Ijrras*.

[45] Fatriansyah J. F., Rizqillah R. K., Yandi M. Y. (2022). Molecular docking and molecular dynamics simulation of fisetin, galangin, hesperetin, hesperidin, myricetin, and naringenin against polymerase of dengue virus. *Journal of Tropical Medicine*.

[46] Kala C. P. (2012). Leaf juice of carica papaya L.: a remedy of dengue fever. *Medicinal & Aromatic Plants*.

[47] Gadhwal A. K., Ankit B. S., Chahar C., Tantia P., Sirohi P., Agrawal R. P. (2016). Effect of carica papaya leaf extract capsule on platelet count in patients of dengue fever with thrombocytopenia. *Journal of the Association of Physicians of India*.

[48] Rajapakse S., de Silva N. L., Weeratunga P., Rodrigo C., Sigera C., Fernando S. D. (2019). Carica papaya extract in dengue: a systematic review and meta-analysis. *BMC Complementary and Alternative Medicine*.

[49] Norahmad N. A., Mohd Abd Razak M. R., Mohmad Misnan N. (2019). Effect of freeze-dried Carica papaya leaf juice on inflammatory cytokines production during dengue virus infection in AG129 mice. *BMC Complementary and Alternative Medicine*.

[50] Mishra S., Aeri V., Gaur P. K., Jachak S. M (2014). Phytochemical, therapeutic, and ethnopharmacological overview for a traditionally important herb:Boerhavia diffusalinn. *BioMed Research International*.

[51] Adeku E., Osundahunsi O. F., Malomo S. A., Asasile I. I., Owolabi O. M., Oyewole G. (2022). Phytochemical constituents and assessment of crude extracts from Boerhavia diffusa L. and Lonchocarpus sericeus (Poir.) Kunth ex DC. leaves for antioxidant and antibacterial activities. *Measurement: Food*.

[52] Apu A. S., Liza M. S., Jamaluddin A. T. M. (2012). Phytochemical screening and in vitro bioactivities of the extracts of aerial part of Boerhavia diffusa Linn. *Asian Pacific Journal of Tropical Biomedicine*.

[53] Bharati P., Sinha R. (2012). Study the effect of tinospora cardifolia (wild) miers and boerhaavia diffusia linn on dengue. *International Journal of Ayurvedic and Herbal Medicine*.

[54] Bourjot M., Leyssen P., Eydoux C. (2012). Flacourtosides A-F, phenolic glycosides isolated from flacourtia ramontchi. *Journal of Natural Products*.

[55] Dutta P. (2015). Pharmacognostical evaluation and preliminary phytochemical analysis of Alternanthera philoxeroides. *International Journal of Medicine and Pharmaceutical Research*.

[56] Jiang W., Luo X., Kuang S. (2005). Effects of alternanthera philoxe-roides griseb against deirus invitro. *Journal First Army Medical University*.

[57] Alzohairy M. A. (2016). Therapeutics role of azadirachta indica (neem) and their active constituents in diseases preven-tion and treatment. *Evidence-based Complementary and Alternative Medicine*.

[58] Parida M. M., Upadhyay C., Pandya G., Jana A. M. (2002). Inhibitory potential of neem (Azadirachta indica Juss) leaves on dengue virus type-2 replication. *Journal of Ethnopharmacology*.

[59] Lavanya P., Ramaiah S., Anbarasu A. (2015). Computational analysis reveal inhibitory action of nimbin against dengue viral envelope protein. *Virus Dis*.

[60] Dwivedi V. D., Tripathi I. P., Mishra S. K. (2016). In silico evaluation of inhibitory potential of triterpenoids from Azadirachta indica against therapeutic target of dengue virus, NS2B-NS3 protease. *Journal of Vector Borne Diseases*.

[61] Brandao G. C., Kroon E., Souza D. (2013). Chemistry and antiviral activity of arrabidaea pulchra (Bignoniaceae). *Molecules*.

[62] Brandão G., Kroon E., Dos Santos J., Stehmann J., Lombardi J., Braga de Oliveira A. (2010). Antiviral activity of bignoniaceae species occurring in the state of minas gerais (Brazil): Part 1. *Letters in Applied Microbiology*.

[63] Hossain M. D., Urbi Z., Sule A., Rahman K. M. (2014). Andrographis Paniculata (Burm. f.) Wall. Ex Nees: A Review of Ethnobotany, Phytochemistry, and Pharmacology. *The Scientific World Journal*.

[64] Kaushik S., Dar L., Kaushik S., Yadav J. P. (2021). Identification and characterization of new potent inhibitors of dengue virus NS5 proteinase from Andrographis paniculata supercritical extracts on in animal cell culture and in silico approaches. *Journal of Ethnopharmacology*.

[65] Panraksa P., Ramphan S., Khongwichit S., Smith D. R. (2017). Activity of andrographolide against dengue virus. *Antiviral Research*.

[66] Karlowicz-Bodalska K., Han S., Freier J., Smolenski M., Bodalska A. (2017). Curcuma longa as medicinal herb in the treatment of diabetic complications. *Acta Poloniae Pharmaceutica*.

[67] Sayantani Chanda T. V. (2019). Ramachandra. Phytochemical and pharmacological importance of turmeric (curcuma longa): a review. *Research & Reviews: A Journal of Pharmacology*.

[68] Padilla-S L., Rodríguez A., Gonzales M. M., Gallego-G J. C., Castaño-O J. C. (2014). Inhibitory effects of curcumin on dengue virus type 2 infected cells in vitro. *Archives of Virology*.

[69] Balasubramanian A., Pilankatta R., Teramoto T. (2019). Inhibition of dengue virus by curcuminoids. *Antiviral Research*.

[70] Tan S. K., Pippen R., Yusof R., Rahman N. A., Ibrahim H., Khalid N. Z. (2006). Screening of selected Zingiberaceae extracts for dengue-2 virus protease inhibitory activities. *Sunway Academic Journal*.

[71] Ichsyani M., Ridhanya A., Risanti M. (2017). Antiviral effects of Curcuma longa L. against dengue virus in vitro and in vivo. *IOP Conference Series: Earth and Environmental Science*.

[72] Kaur J., Nafees S., Anwar M., Akhtar J., Anjum N. (2017). Glycyrrhiza glabra (Licorice) and gymnema sylvestre (gurmar). *Herbs, Shrubs, and Trees of Potential Medicinal Benefits*.

[73] Al-Snafi A. E. (2018). Glycyrrhiza glabra: a phytochemical and pharmacological review. *IOSR Journal of Pharmacy*.

[74] N Powers C., N Setzer W. (2016). An In-silico investigation of phy-tochemicals as antiviral agents against dengue fever. *Combinatorial Chemistry & High Throughput Screening*.

[75] Fiore C., Eisenhut M., Krausse R. (2008). Antiviral effects of Glycyrrhiza species. *Phytotherapy Research*.

[76] Rahman M. M., Biswas S., Islam K. J. (2021). Antiviral phytochemicals as potent inhibitors against NS3 protease of dengue virus. *Computers in Biology and Medicine*.

[77] Eng-Chong T., Yean-Kee L., Chin-Fei C. (2012). Boesen-bergia rotunda: from ethnomedicine to drug discovery. *Evidence-based Complementary and Alternative Medicine*.

[78] Jiraungkoorskul W., Ongwisespaiboon O. (2017). Fingerroot, Boesenbergia rotunda and its aphrodisiac activity. *Pharmacognosy Reviews*.

[79] Kanjanasirirat P., Suksatu A., Manopwisedjaroen S. (2020). High-content screening of Thai medicinal plants reveals Boesenbergia rotunda extract and its component Panduratin A as anti-SARS-CoV-2 agents. *Scientific Reports*.

[80] Bhattarai B. R., Adhikari B., Basnet S. (2022). In silico elucidation of potent inhibitors from natural products for nonstructural proteins of dengue virus. *Journal of Chemistry*.

[81] Chowdhury M. A. R., Haq M. M. (2017). Phyto-chemical and pharmacological activity of myristica fra-grans houtt (myristicaceae). *International journal of pharmacology and toxicology*.

[82] Rosmalena R., Elya B., Dewi B. E. (2019). The antiviral effect of Indonesian medicinal plant extracts against dengue virus in vitro and in silico. *Pathogens*.

[83] Manojj D., Yasasve M., Kanmani K., Sai Ramesh A. (2020). In vitro cytotoxicity study and anti-brucella activity of tarenna asi-atica (L). *South African Journal of Botany*.

[84] Pratheeba T., Taranath V., Sai Gopal D. V. R., Natarajan D. (2019). Antidengue potential of leaf extracts of pavetta tomentosa and tarenna asiatica (rubiaceae) against dengue virus and its vector aedes aegypti (diptera: culicidae). *Heliyon*.

[85] Cui J., Hu W., Cai Z. (2010). Newmedicinal properties of mangostins: analgesic activity andpharmacological characterization of active ingredients from the fruit hull of garcinia mangostana L. *Pharmacology Biochemistry and Behavior*.

[86] Obolskiy D., Pischel I., Siriwatanametanon N., Heinrich M. (2009). Garcinia mangostana L.: a phytochemical and pharmacological review. *Phytotherapy Research*.

[87] Gunathilaka N., Wijebandara Y., Amerasinghe D., Udayanga L., Muhandiramlage T. P. (2022). Larvicidal activity of the pericarp extract of Garcinia mangostana against dengue vector. *Aedes aegypti in Sri Lanka*.

[88] Perera S. D., Jayawardena U. A., Jayasinghe C. D. (2018). Potential Useof euphorbia hirta for dengue: a systematic review of scien-tific evidence. *Journal of Tropical Medicine*.

[89] Ghosh P., Ghosh C., Das S., Das C., Mandal S., Chatterjee S. (2019). Botanical description phytochemical constituents and pharmacological properties of Euphorbia hirta Linn . a review. *International Journal of Health Sciences & Research*.

[90] Berg C., Corner E. J. H. (2005). *Moraceae Flora Malesiana I*.

[91] Ashraf K., Haque M. R., Amir M. (2021). An overview of phytochemical and biological activities: Ficus deltoidea Jack and other Ficus spp. *Journal of Pharmacy and BioAllied Sciences*.

[92] Joseph B., Raj S. J. (2010). Phytopharmacological and phytochemical properties of three Ficus species-an overview. *International Journal of Pharma Bio Sciences*.

[93] Kumar A., Sandeep D., Tomer V., Gat Y., Kumar V. (2018). Ficus religiosa: a wholesome medicinal tree. *Journal of Pharmacognosy and Phytochemistry*.

[94] Huang N. C., Hung W. T., Tsai W. L. (2017). Ficus septica plant extracts for treating Dengue virus in vitro. *PeerJ*.

[95] Rahman S. M. M., Atikullah M., Islam M. N. (2019). Anti-inflammatory, antinociceptive and antidiarrhoeal activitiesof methanol and ethyl acetate extract of hemigraphis alter-nata leaves in mice. *Clinical Phytoscience*.

[96] Rothan H. A., Zulqarnain M., Ammar Y. A., Tan E. C., Rahman N. A., Yusof R. (2014). Screening of antiviral activities in medicinal plants extracts against dengue virus using dengue NS2B-NS3 protease assay. *Tropical Biomedicine*.

[97] Jamshidi N., Cohen M. M. (2017). The clinical efficacy and safetyof tulsi in humans: a systematic review of the literature. *Evidence-based Complementary and Alternative Medicine*.

[98] Rahman S., Islam R., Kamruzzaman M., Alam K., Jamal A. H. M. (2011). Ocimum sanctum L.: a review of phytochemical and pharmacological profile. *American journal of drug discovery and Development*.

[99] Singh D., Chaudhuri P. K. (2018). A review on phytochemical and pharmacological properties of Holy basil (Ocimum sanctum L.). *Industrial Crops and Products*.

[100] Ling A. P. K., Khoo B. F., Seah C. H. Inhibitory activities of methanol extracts of Andrographis paniculata and Ocimum sanctum against dengue-1 virus.

[101] Yi H., Chen Y., Liu J. (2016). Extraction and separation of active ingredients in schisan-dra chinensis (turcz.) baill and the study of their antifungal effects. *PLoS One*.

[102] Yu J. S., Wu Y. H., Tseng C. K. (2017). Schisandrin A inhibits dengue viral replication via upregulating antiviral interferon responses through STAT signaling pathway. *Scientific Reports*.

[103] Teixeira R. R., Pereira W. L., Oliveira A. F. C. D. S. (2014). Natural products as source of potential dengue antivirals. *Molecules*.

[104] Chai X. Y., Ren H. Y., Xu Z. R. (2009). Investigation of two Flacourtiaceae plants: bennettiodendron leprosipes and Flacourtia ramontchi. *Planta Medica*.

[105] Allard P. M., Dau E. T. H., Eydoux C. (2011). Alkylated flavanones from the bark of Cryptocarya chartacea as dengue virus NS5 polymerase inhibitors. *Journal of Natural Products*.

[106] Chiow K. H., Phoon M. C., Putti T., Tan B. K., Chow V. T. (2016). Evaluation of antiviral activities of Houttuynia cordata Thunb. extract, quercetin, quercetrin and cinanserin on murine coronavirus and dengue virus infection. *Asian Pacific journal of tropical medicine*.

[107] Akram M., Adetunji C. O., Egbuna C. (2021). Dengue fever: a brief overview and insights into the potential applicability of phytochemicals in its management. Neglected tropical diseases and phytochemicals in drug discovery.

[108] Leardkamolkarn V., Sirigulpanit W., Phurimsak C., Kumkate S., Himakoun L., Sripanidkulchai B. (2012). The inhibitory actions of Houttuynia cordata aqueous extract on dengue virus and dengue‐infected cells. *Journal of Food Biochemistry*.

[109] Hemalatha S., Kumar M., Prasad S. (2014). A current update on the phytopharmacological aspects of Houttuynia cordata Thunb. *Pharmacognosy Reviews*.

[110] Chen X., Wang Z., Yang Z. (2011). Houttuynia cordata blocks HSV infection through inhibition of NF-*κ*B activation. *Antiviral Research*.

[111] Wang Z. L., Wang S., KuangHu Y., QiaoYe M. (2018). A comprehensive review on phytochemistry, pharmacology, and flavonoid biosynthesis of Scutellaria baicalensis. *Pharmaceutical Biology*.

[112] Wang Z. L., Wang S., Kuang Y., Hu Z. M., Qiao X., Ye M. (2018). A comprehensive review on phytochemistry, pharmacology and flavonoid biosynthesis of Scutellariabaicalensis. *Pharmaceutical Biology*.

[113] Moghaddam E., Teoh B. T., Sam S. S. B. (2014). Baicalin, a metabolite of baicalein with antiviral activity against dengue virus. *Scientific Reports*.

[114] Zandi K., Teoh B., Sam S., Wong P., Mustafa M. R., Abubakar S. (2012). Novel antiviral activity of baicalein against dengue virus. *BMC Complementary and Alternative Medicine*.

[115] Zhao Q., Chen X. Y., Martin C. (2016). Scutellaria baicalensis, the golden herb from the garden of Chinese medicinal plants. *Science Bulletin*.

[116] Zandi K., Lim T. H., Rahim N. A. (2013). Extract of Scutellaria baicalensis inhibits dengue virus replication. *BMC Complementary and Alternative Medicine*.

[117] Simoes L. R., Maciel G. M., Brandao G. C., Filho J. D., Oliveira A. B., Castilho R. O. (2013). Chemical constituents of distictella elongata (vahl) urb.(bignoniaceae). *Anais da Academia Brasileira de Ciências*.

[118] Simoes L., Maciel G., Brandao G., Kroon E., Castilho R., Oliveira A. (2011). Antiviral activity of distictella elongata (vahl) urb. (bignoniaceae), a potentially useful source of anti-dengue drugs from the state of minas gerais, Brazil. *Letters in Applied Microbiology*.

[119] Trejo-Avila L. M., Morales-Martínez M. E., Ricque-Marie D. (2014). In vitro anti-canine distemper virus activity of fucoidan extracted from the brown alga Cladosiphon okamuranus. *VirusDisease*.

[120] Hidari K. I., Takahashi N., Arihara M., Nagaoka M., Mo-rita K., Suzuki T. (2008). Structure and anti-dengue virus activity of sulfated polysaccharide from a marine alga. *Biochemical and Biophysical Research Communications*.

[121] Talarico L. B., Zibetti R. G., Faria P. C. (2004). Anti-herpes simplex virus activity of sulfated galactans from the red seaweeds Gymnogongrus griffithsiae and Cryptonemia crenulata. *International Journal of Biological Macromolecules*.

[122] Talarico L. B., Pujol C. A., Zibetti R. G. M. (2005). The antiviral activity of sulfated polysaccharides against dengue virus is dependent on virus serotype and host cell. *Antiviral Research*.

[123] Sajeesh T., Parimelazhagan T. (2014). Analgesic, anti-inflammatory, and GC-MS studies on castanospermum australe A.Cunn. & C, Fraser Ex hook. *The Scientific World Journal*.

[124] Whitby K., Pierson T. C., Geiss B. (2005). Castanospermine, a potent inhibitor of dengue virus infection in vitro and in vivo. *Journal of Virology*.

[125] Amresh G., Singh P. N., Rao C. (2007). Antinociceptive and antiarthritic activity of cissampelos pareira roots. *Journal of Ethnopharmacology*.

[126] Sood R., Raut R., Tyagi P. (2015). Cissampelos pareira Linn: Natural Source of Potent Antiviral Activity against All Four Dengue Virus Serotypes. *PLOS Neglected Tropical Diseases*.

[127] Tarasuk M., Songprakhon P., Chimma P., Sratongno P., Na-Bangchang K., Yenchitsomanus Pt (2017). Alpha-Mangostininhibits both dengue virus production and cytokine/chemo kine expression. *Virus Research*.

[128] Panraksa P., Ramphan S., Khongwichit S., Smith D. R. (2017). Activ-ity of andrographolide against dengue virus. *Antiviral Research*.

[129] Parida M. M., Upadhyay C., Pandya G., Jana A. M. (2002). Inhibitory Potential of neem (azadirachta indica juss) leaves on dengue virus type-2 replication. *Journal of Ethnopharmacology*.

[130] Frimayanti N., Zain S. M., Lee V. S., Wahab H. A., Yusof R., Abd Rahman N. (2012). Fragment-based molecular design of new com-petitive dengue Den2 Ns2b/Ns3 inhibitors from the com-ponents of fingerroot (boesenbergia rotunda). *In Silico Biology*.

[131] Rosmalena R., Elya B., Dewi B. E. (2019). The antiviral effect of Indonesian medicinal plant extracts against dengue virus in vitro and in silico. *Pathogens*.

[132] Guzman G., Dacanay A. T., Andaya B. A., Alejandro G. J. (2016 Jun). Ethnopharmacological studies on the uses of Euphorbia hirta in the treatment of dengue in selected indigenous communities in Pangasinan (Philippines). *Journal of intercultural ethnopharmacology*.

[133] Tennyson S., Samraj D. A., Jeyasundar D., Chalieu K. (2013). Larvicidal efficacy of plant oils against the dengue vector Aedes aegypti (L.)(Diptera: Culicidae). *Middle-East Journal of Scientific Research*.

[134] Yu J. S., Wu Y. H., Tseng C. K. (2017). Schisandrin A inhibits dengue viral replication via upregulating antiviral interferon responses through STAT signaling pathway. *Scientific Reports*.

[135] Chiow K. H., Phoon M. C., Putti T., Tan B. K. H., Chow V. T. (2016). Evaluation of antiviral activities of Houttuynia cordata Thunb. extract, quercetin, quercetrin and cinanserin on murine coronavirus and dengue virus infection. *Asian Pacific Journal of Tropical Medicine*.

